# ICD-11 Personality Disorders: Utility and Implications of the New Model

**DOI:** 10.3389/fpsyt.2021.655548

**Published:** 2021-05-10

**Authors:** Roger T. Mulder

**Affiliations:** Department of Psychological Medicine, University of Otago, Christchurch, New Zealand

**Keywords:** personality disorder, classification, domains, diagnosis, ICD-11

## Abstract

The ICD-11 classification of personality disorders represents a paradigm shift in diagnosis. This was felt necessary because previous personality disorder classifications had major problems. These included unnecessary complexity, inconsistency with data on normal personality traits, and minimal consideration of severity despite this being shown to be the major predictor of outcome. The ICD-11 classification abolishes all categories of personality disorder except for a general description of personality disorder. This diagnosis can be further specified as “mild,” “moderate,” or “severe.” Patient behavior can be described using one or more of five personality trait domains; negative affectivity, dissociality, anankastia, detachment, and disinhibition. Clinicians may also specify a borderline pattern qualifier. The ICD-11 shows considerable alignment with the DSM-5 Alternative Model for Personality Disorders. Early evidence around the reliability and validity of the new model appear promising, although at present there is still limited specific evidence due to the model being so recently finalized. However, for the model to be successful, it needs to be embraced by clinicians and used widely in normal clinical practice.

## Introduction

When the ICD-11 working group for the revision of the classification of personality disorders was established in 2010 there was a great deal of dissatisfaction with the current ICD-10 (1) and DSM IV (2) classifications. First, the system was too complex with around 80 criteria, some of which overlapped, and 10 separate categories based on no coherent model or theory. The descriptions appear to have evolved from historical precedents, clinical experience, and committee consensus. Some categories had their origins in Galen's temperaments described over 2,000 years ago, while others, such as Borderline Personality Disorder, appeared in 1980. Clinicians responded logically; they largely ignored the whole concept of personality disorder, resulting in rates of diagnoses being less than one quarter of that reported in systematic reviews (3). When clinicians did make a personality disorder diagnosis, they generally used two of the 10 official categories, borderline and antisocial, as well as the catch all “personality disorders not otherwise specified” (PD NOS). In addition, the complexity of personality disorder nosology resulted in any interest being confined to the specialist few, with the general clinician becoming even less involved.

Second, the classification was inconsistent with what data was available, with most evidence suggesting personality abnormality was distributed along a dimension (4). These dimensional constructs were similar to dimensions of personality which have been reported in the general population. Probably not surprisingly, normal and abnormal personality are, at least to some extent, related to each other (5). The question of whether using a dimensional model of personality to understand personality disorders can work has been subject to significant scrutiny. Generally, there is empirical support for such a dimensional conceptualization. Four meta-analyses using a total of 52 independent samples involving 13,640 individuals concluded that personality pathology can be adequately represented as constellations of extreme scores on normal personality models, most notably the five-factor model (6). However, a dimensional approach has been slow to be accepted by those involved in the classification of personality pathology. While a number of authors had suggested that a dimensional approach to personality disorders was most appropriate by the early 90s (7, 8), little agreement existed on what direction progress should take.

Third, there is consistent evidence that the severity rather than the type of personality pathology is the major predictor of the individual's suffering and dysfunction (9). The total number of diagnosed personality disorders or the number of traits explains more variability in functioning than specific personality disorders alone (10). Those with more severe personality disturbance are more likely to self-harm (11), to have a greater degree of co-morbidity and suicide risk (12), and to have a higher risk of treatment drop-out (13). The argument can also be made that prioritizing severity helps re-focus on the core management around self and interpersonal difficulties rather than emphasizing behavioral descriptions.

## Discussion

### The ICD-11 Proposal

#### Severity of Personality Disturbance

Therefore, it is not surprising that when the ICD-11 personality disorder classification committee met in 2010, they felt that a paradigm shift was necessary. Changing the symptoms within categories or changing the number of categories would be insufficient. Tinkering around the margins would not address the fundamental problems with the ICD-10 classification system. Led by Peter Tyrer, the initial proposal for the ICD-11 classification set out to abolish all categories of personality disorder except for the general description of a personality disorder. Personality disorder was conceptualized along a dimension of severity. So, to qualify for a diagnosis of personality disorder, general diagnostic features such as problems in interpersonal relationships and impaired functioning must be present. The diagnosis could then be further specified as “mild,” “moderate,” or “severe” based upon descriptions of degrees of severity. Assessment of severity was based on the prominence of the abnormal traits and their impact on the individual's social and occupational functioning, as well as the risk to themselves or others. Although some description details have been changed since the initial formulation, particularly an increased emphasis on aspects of self-functioning, the fundamental concept has remained and is now embedded in ICD-11 [the final definitions of severity are outlined in the World Health Organization's ICD-11 website (2018)].

#### Personality Difficulty

What is new, however, is the concept of personality difficulty which, while not a disorder, is described as “problems associated with interpersonal interactions.” More specifically, the definition refers to “pronounced personality characteristics that may affect treatment or health services but do not rise to the level of severity to merit a diagnosis of personality disorder.” In contrast to personality disorders, personality difficulty is manifested in “cognitive and emotional experience and expression only intermittently or at low intensity” (14). There is some existing data suggesting that the concept of personality difficulty may be clinically useful. The UK National Morbidity Survey assessed 8,400 individuals for personality status and mental health. When personality difficulty was defined as a score of one operational criterion less than personality disorder on a SCID-II nearly half the respondents (48.3%) fulfilled criteria. Having this diagnosis was not trivial; they were significantly more likely to consult their general practitioners, be admitted to a mental hospital, attend a community medical center or see a mental health worker (15). Some are concerned that the term will lead to over-medicalisation of behavior and become referred to as a diagnosis even though it is explicitly stated that it is not one. However, the concept has potential use in the general medical and general practice sphere where clinicians often note that patients with the same physical diagnoses may require quite different treatment approaches due to personality quirks.

#### Personality Trait Domains

With regard to the descriptions of personality pathology, the ICD-11 proposal was quite radical. Rather than compromising by retaining some diagnostic categories, as the DSM-5 proposal did, the model included replacement of all ICD-10 diagnostic categories. This significant shift toward a dimensional descriptive framework was guided by literature review (3) and subsequently informed by field trails (16–18). The field trails produced mixed findings; while the anankastia and detached domains appeared reasonably robust, the negative affectivity and dissocial were less so. Two of the analyses suggested that five trait domains were a better fit for the data (17). While the initial model had four traits which were (a) negative affectivity, (b) dissociality, (c) anankastia, and (d) detachment, after considerable debate, disinhibition was later added. As we shall see, its inclusion may have led to as many problems as it seemed to solve.

Despite being derived independently, the five personality trait domains are similar to the DSM-5 Alternative Model for Personality Disorders (AMPD); the major difference being the anankastia domain in ICD-11 vs. the DSM-5 psychotic domain. This similarity is reassuring as well as allowing DSM AMPD domains to be translated to ICD-11 domains, notably by Bach et al. (19, 20).

The proposal received mixed feedback; most clinicians appeared to like it, but some personality disorder researchers considered it too minimalist and feared dropping established categories (21–23). While most experts believed that personality pathology was dimensional in nature, a majority preferred a hybrid model (a mixture of dimension and categories) (4). Just before the WHO final approval of the ICD-11 representatives from the European, International, and North American Societies for the Study of Personality Disorders expressed their strong concerns (24). The central issue was the loss of Borderline Personality Disorder. Further discussion between some ICD-11 working group members and representatives from the Societies reached a compromise (25, 26). This included specifying a borderline pattern qualifier (which is very similar to the DSM-5 Borderline Personality Disorder diagnosis) and the general definition having more focus on self as well as interpersonal functioning. For a more detailed overview of the process see Tyrer et al. (27).

### The Validity and Utility of ICD-11 Personality Disorders

#### Severity of Personality Disorder

One problem with such a radical change in the classification system is that the findings of previous studies cannot always be directly translated into the new system. There is, therefore, limited specific evidence of the utility and validity of the ICD-11 classification at this time. Fortunately, the DSM-5 AMPD, which despite not being the official DSM-5 classification has been the most used in recent personality disorder research studies (28), and can be reasonably translated into the ICD-11 system. The similarities are important in allowing the large body of work supporting the DSM-5 AMPD model to be generalized to the ICD-11 personality disorder model. Table 1 shows the alignment between the two models.

**Table 1:** Alignment between ICD-11 and DSM-5 alternative model for personality disorders models.

**ICD-11 severity of personality dysfunction**	**DSM-5 criterion A: level of personality functioning**
None	0) No impairment (Healthy Functioning)
Personality difficulty	1) Some impairment
Mild personality disorder	2) Moderate impairment
Moderate personality disorder	3) Severe impairment
Severe personality disorder	4) Extreme impairment
**ICD-11 trait domain qualifiers**	**DSM-5 criterion B: trait domains**
Negative affectivity	Negative affectivity
Detachment	Detachment
Disinhibition	Disinhibition
Dissociality	Antagonism
Anankastia	(Rigid perfectionism)
(Schizotypal disorder)	Psychoticism
**Continuity with clinical practice**	**Hybrid types**
Borderline pattern qualifier	Antisocial, Avoidant, Borderline, Narcissistic, Obsessive-Compulsive, Schizotypal, Trait-Specified

While the ICD-11 levels of severity compliment the DSM-5 impairment levels of personality functioning, they also add to them. ICD-11 provides a separate list of explicit emotional, cognitive, and behavioral manifestations to help determine the severity of an individual personality disorder (29). Therefore, severe personality disorder may include stress-related distortions in an individual's situational and interpersonal beliefs which could lead to psychotic-like perceptions. The system also explicitly includes risk of harm to self and others as part of determining personality disorder severity. Finally, the model includes the complexity and pervasiveness of the disturbance as a determinant of severity. Severe personality disorder by definition, affects most, if not all, areas of personality functioning, while mild personality disorder will only involve some areas.

#### Personality Trait Domains

The ICD-11 trait domains also have the advantage of being conceptually aligned with the AMPD trait domains. There is now consistent evidence that both systems converge and largely capture the same descriptive features (20, 30, 31) so that the extensive research on AMPD traits (with the obvious exception of psychoticism) may be generalized to ICD-11 trait domains when it comes to clinical information and guidelines. Bach et al. (19) developed an algorithm to delineate the five ICD-11 trait domains (including anankastia) using the well-established Personality Inventory for DSM-5 (PiD) (32). The ICD-11 five factor structure was supported across US and Danish data. Other populations, including Iranian and Brazilian samples, have replicated the five factor structure (33, 34). Sellbom et al. (35) reported that the ICD-11 traits demonstrated expected associations with categorical personality disorder symptom scores and five factor traits in a sample of Canadian psychiatric patients.

Oltmanns and Widiger (30) produced a scale specifically developed for the ICD-11 trait domains—the Personality Inventory for ICD-11 (PiCD). Evaluation in several studies generally supports the validity of the ICD-11 trait domains. Importantly, the domains converged predictably with external criteria, notably the five factor model. As predicted, negative affectivity is associated with neuroticism, detachment with low extraversion, dissociality with low agreeableness, anankastia with high orderliness, and disinhibition with low orderliness (36). Crego and Widiger (37) used a sample of 323 patients in mental health treatment and reported meaningful and expected convergence of the ICD-11 domains with SNAP and DAPP-BQ. The anankastia domain was noted to have strong predictable relationships with compulsivity measures on both scales, which were not present in the PID-5.

There is limited evidence on the utility of the trait domains. A study by Hansen et al. (38) reported that clinicians rated the ICD-11 slightly more useful than the ICD-10 classification. The final definitions of the ICD trait domains were produced in 2018 so specific evidence of their utility is not available yet. However, the predicted relationships with other measures of personality suggest they may prove useful in clinical practice.

#### The Anankastia Domain Qualifier

This trait domain has been discussed more than the other domains for two reasons. The first is that it is not a domain in DSM-5 AMPD model. The second is that some studies suggest it is not a separate domain but part of a bipolar disinhibition-anankastia factor. Regarding the first reason; compulsivity, which is roughly equal to anankastia, was originally proposed as a distinct domain in the DSM-5 trait model (39). It was abandoned for reasons which are not clear. The generated list of 37 facets for the DSM-5 AMPD included (lack of) orderliness, (lack of) perfectionism, and (lack of) rigidity, which are aligned with an anankastia or compulsivity domain. However, these facets were subsumed into the disinhibited domain (32). Our review (3) found that anankastia or something similar was reported in most analyses which looked for it, while a disinhibition dimension was less consistently found.

That said, there are legitimate concerns about the relationship of the disinhibited and anankastia domains in the ICD-11 classification. Two studies have reported a four factor solution including a bipolar disinhibition-anankastia domain (30, 31). However, other studies support a five factor solution in which anankastia and disinhibition are two distinct domains (17, 19, 35). The clinical reality may be that complex personality disorder patterns can be characterized by both disinhibition and anankastia (40).

Given the consistency of an anankastia domain, or something like it, in the literature, it may be that the disinhibited domain deserves more scrutiny. Disinhibition may be a more general trait which renders personality pathology, and possibly other psychopathology, more obvious across a variety of disorders. The behaviors associated with rigid perfectionism exist and it is not intuitive for clinicians to diagnose “low disinhibition” or “lack of disinhibition” as a personality description. In the end, clinical utility will probably determine whether both the anankastia and disinhibited domains are helpful, or whether one should be dropped.

#### Borderline Pattern Qualifier

The ICD-11 classification allows clinicians to specify a borderline pattern qualifier, which essentially consists of the nine DSM-5 diagnostic features. This coding was a pragmatic solution to objections from many senior researchers around the loss of this historically important construct (24). Nevertheless, the ICD-11 working group felt that the severity and trait model could fully account for the borderline pattern (27). The borderline pattern qualifier appears closely linked to the overall severity of the personality pathology and may be a redundant addition to the classification (41). It is hoped that research will reveal whether the borderline pattern qualifier provides useful or distinct information beyond that provided by the trait domains (26).

## Conclusion

The ICD-11 personality disorder classification is now official and will be required to be used in many countries from January 2022. It is hoped that its use will lead to greater understanding of the concept of personality disorder and better clinical care. It moves from an unnecessarily complicated classification system, most categories of which were never used, to a simpler, more evidence-based model. However, the radical change and novelty in the new classification mean that most of the evidence around utility and validity are indirect and based on other measures of personality pathology, since the new model was not fully described until 2018.

The ICD-11 severity diagnosis relies on research consistently indicating that the level of personality functioning and personality disorder complexity are the most important clinical and prognostic factors. The ICD-11 personality trait domain qualifiers are an attempt to distill personality descriptions into meaningful dimensions. Their empirical foundation is largely shared with the DSM-5 AMPD model and other well-established models describing personality, including the five factor model. The trait domain qualifiers can now be evaluated using different measures and algorithms as well as specifically developed instruments.

The changes in ICD-11 personality disorder classification represent a paradigm shift in diagnosis. But for it to be successful, it needs to be embraced by clinicians and used in practice in a way that has never been done before. To aid this, brief self-report questionnaires and clinical interviews are being developed. If further studies show, as I believe, that personality can now be recorded reliably with relative ease, and is useful in planning and predicting the outcome of treatment in most mental disorders, then clinicians may realize the advantage of bringing personality disorders to the forefront of their thinking, instead of the afterthought so often in present day clinical practice.

## Author Contributions

The author confirms being the sole contributor of this work and has approved it for publication.

## Conflict of Interest

RM was a member of the ICD-11 Personality Disorder Classification Committee.
